# Supplementary material for: An Assassin’s Secret: Multifunctional Cytotoxic Compounds in the Predation Venom of the Assassin Bug Psytalla horrida (Reduviidae, Hemiptera)
Source: Toxins (Basel). 2023 Apr 20;15(4):302. doi: 10.3390/toxins15040302 (PMC10144120; doi:10.3390/toxins15040302)
Supplement: Supplementary file 1 [file toxins-15-00302-s001.zip › toxins-2345474-supplementary.pdf]

# Supplemental Materials: An Assassin's Secret: Multifunctional Cytotoxic Compounds in the Predation Venom of the Assassin Bug *Psytalla horrida* (Reduviidae, Hemiptera)

Maike Laura Fischer, Benjamin Fabian, Yannick Pauchet, Natalie Wielsch, Silke Sachse, Andreas Vilcinskas and Heiko Vogel

**Table S1.** Summary of the tests used to statistically analyze the results from the bioassays. .

| | Assay | | Test | P | Test |
|---|---|---|---|---|---|
| **Fractions** | Bacterial growth inhibition | | ANOVA | 5.7e-10 | Pairwise t-tests |
| | Cell viability | | Kruskal-Wallis | 1.9e-08 | Pairwise Dunn's tests |
| | Hemolysis | | Kruskal-Wallis | 0.007735 | Pairwise Dunn's tests |
| | Calcium imaging | | Kruskal-Wallis | 9.9e-08 | Pairwise Dunn's tests |
| **Redulysins** | Bacterial growth inhibition | *Escherichia coli* | Kruskal-Wallis | 7.1e-05 | Pairwise Dunn's tests |
| | | *Bacillus subtilis* | ANOVA | 1.9e-11 | Pairwise t-tests |
| | | *Bacillus thuringiensis* | ANOVA | 4.4e-10 | Pairwise t-tests |
| | Cell viability | | Kruskal-Wallis | 6.4e-13 | Pairwise Dunn's tests |
| | Hemolysis | | Kruskal-Wallis | 7.6e-10 | Pairwise Dunn's tests |
| | Calcium imaging | | Kruskal-Wallis | 0.00184 | |
| **Vpf2** | Cell viability | | Kruskal-Wallis | 2.2e-07 | Pairwise Dunn's tests |
| | Calcium imaging | | ANOVA | 0.1866 | |

**Table S2.** Summary of de novo reference transcriptome assembly from Lygus rugulipennis.

| Species | Number of contigs | N50 contig size | BUSCO coverage (Insecta_odb9 database) |
|---|---|---|---|
| *L. rugulipennis* | 66.754 | 1.576 | C:91.4%[S:90.3%,D:1.1%],F:3.9%,M:4.7% |

**Table S3.** *Psytalla horrida* redulysins identified in the genome, PMG transcriptome and proteomes of PMG venom, fraction A and fraction B. The molecular weight of the unprocessed proteins is given in kDa.

| Genome (name) | Molecular weight (kDa) | Transcriptome PMG (name) | Proteome PMG | Proteome Fraction A | Proteome Fraction B |
|---|---|---|---|---|---|
| g1038.t1 | 69.9 | Phor_Comb_C9529 | X | X | |
| g1037.t1 | 55.2 | | | | |
| g1039.t1 | 30.7 | | | | |
| g2020.t1 | 27.1 | Phor_Comb_C34871 | X | | |
| g2021.t1 | 27.2 | Phor_Comb_C46120 | X | | |
| g2022.t1 | 26.7 | Phor_Comb_C52128 | X | | X |
| g2023.t1 | 27.1 | | | | |
| g2565.t1a | 18.7 | Phor_Comb_C25695 | X | | |
| g2565.t1b | 25.1 | Phor_Comb_C44851 | | | |
| g2565.t1c | 19.6 | | | | |
| g17426.t1a | 25.3 | Phor_Comb_C27614 | X | | |
| g17426.t1b | 40.7 | | | | |

**Table S4.** Sequences of synthesized redulysin peptides from *Psytalla horrida* and *Lygus rugulipennis*.

| No | Species | Gene name | | Sequence |
|---|---|---|---|---|
| **1** | *P. horrida* | g1038.t1 domain | 1 | ETWGKMKDHYLNLGKETLAKLKELKEKGQG |
| **2** | | | 2 | DLPYTTKVLLILKETLDKLKAATGEEKEKLIQKIKG |
| **3** | | | 3 | EAWGKMKNHYLNLGKATLAKLNELKENYKT |
| **4** | | | 4 | DPALTAKILLKLKEYLEKLKAAAGKEKEKLILKIKE |
| **5** | | | 5 | KETFGKLKDRWSQLTKAQLQKVIQTLKN |
| **6** | | | 6 | QIIQKYVQKLKDFYEKIKAAVGAKKEELKKRLEE |
| **7** | | | 7 | GWLGKLGKGMKKVGKKFVKKMSSAMKAGCKKGMKMLKD |
| **8** | | g1037.t1 | 1 | EAWGKMKDHYLNLGKATLAKLNELKENYKT |
| **9** | | domain | 5 | GWLGKLGKGMKKVGKKFMKKMSSTMKAGCKKGMKMLKD |
| **10** | | g2020.t1 | | GKVGDWFKKYWKNFKNAMKKLSKEIKEACNKGREFLKK |
| **11** | | g2021.t1 | | GFGDWAQGVWNDAKNVFKKLKKAVKQKCKEGREYLKK |
| **12** | | g2022.t1 | | GKGLDWFKKQWGKMKNSWKKVGAKVKAVFNKGRDFLKK |
| **13** | | g2023.t1 | | GKVGDWFKNQWGKMKNSFKKVGAKMKAVFNKGREFLKK |
| **14** | | g2565.t1a | | KTWTTLKMAVNKLLSRYRKLSKEDFKKILK |
| **15** | | g2565.t1b | | GRWDTFKEKVKIFAKDKKELAKQWAGKFKEWLITTKENAKI-<br>KLKE |
| **16** | | g17426.t1a | | GRKNDKYIGDVIKERLRKLKKSMVEGLRRLKN |
| **17** | | g17426.t1b | | GNKVTSKDPSKPKKKKKFSLKGIGESMKKWAKKGLEILKS |
| **18** | *L. rugulipennis* | Lrug_Comb_C538 | | SKIGSMGKH-<br>VIKQIGKVGLNMLKKGLKVLKSMYYKFGGKRLLKRAKEYLKK |

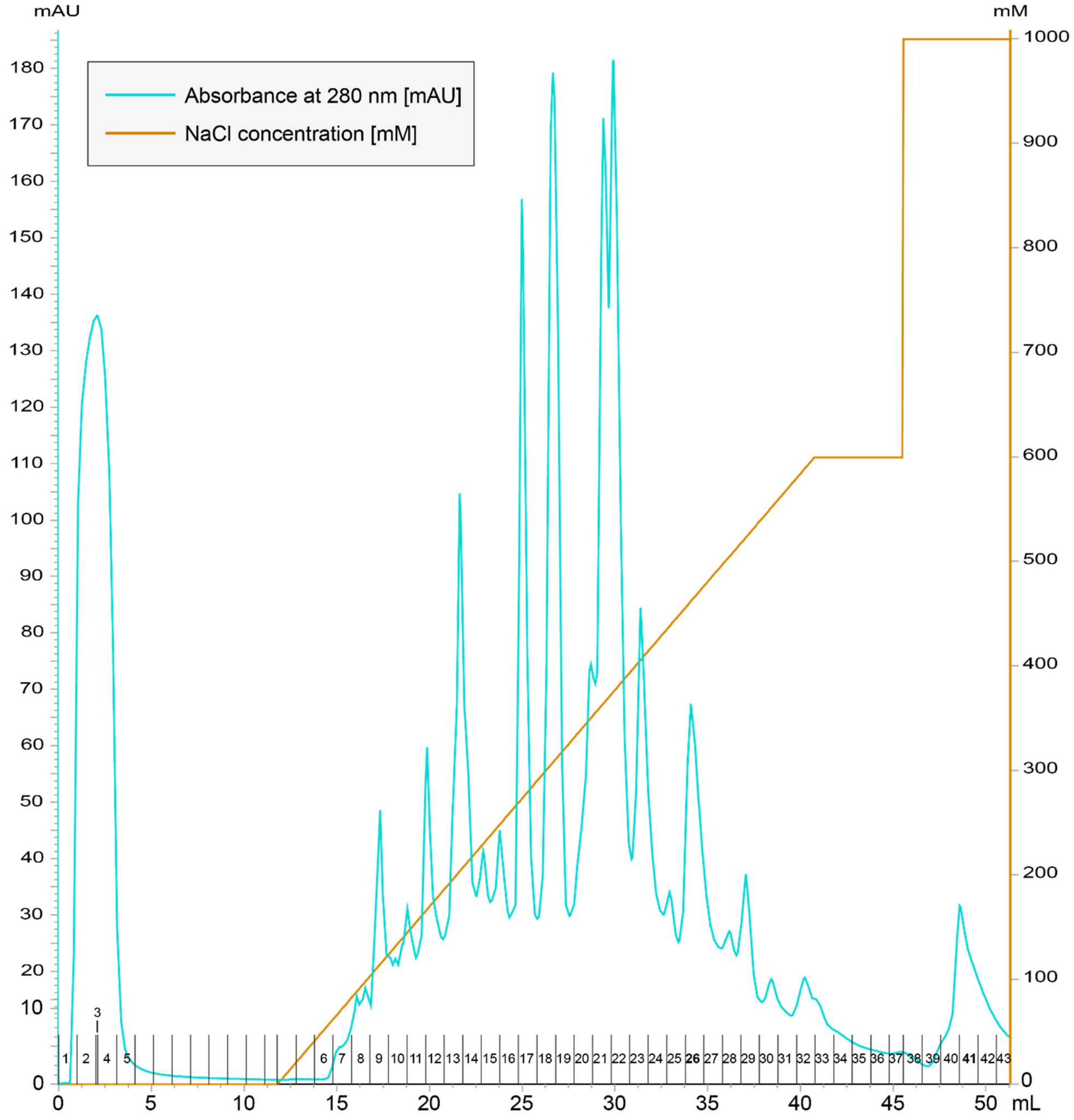


**Figure S1.** Chromatogram of the venom fractionation using cation-exchange chromatography. Crude PMG extract was eluted with 20 mM MES and then fractionated by gradually increasing the NaCl concentration up to 600 mM at a flow rate of 0.5 mL/min. Remaining proteins bound to the column were eluted by adding 20 mM MES containing 1 M NaCl. The blue graph depicts the absorbance of the eluate at 280 nm in mAU. The orange graph shows the NaCl concentration in the course of fractionation in mM. Fractions 1 – 43 that were used for screening are labelled by numbers. The active fractions A (= 26) and B (= 41) are highlighted in bold.

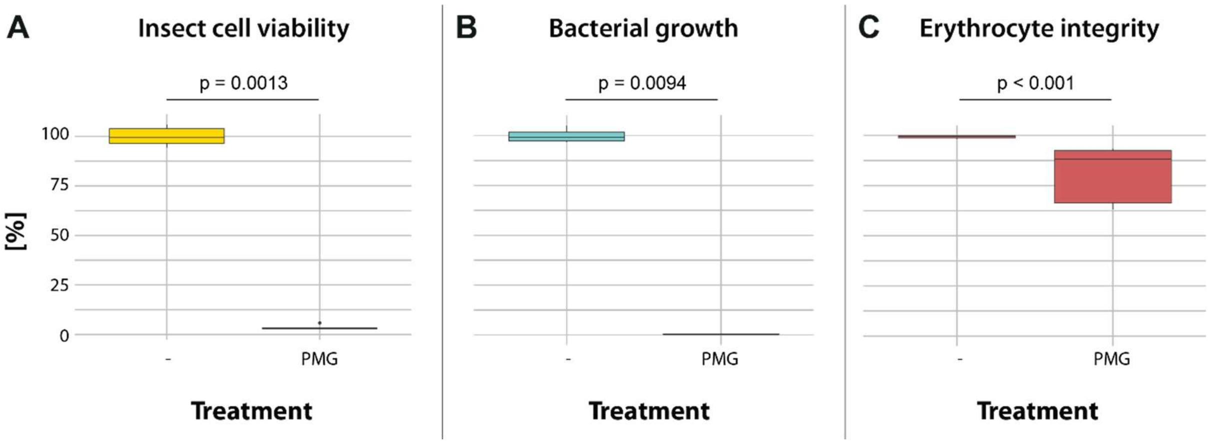


**Figure S2.** Effects of 0.2 mg/ml crude PMG extract on (A) insect cell viability, (B) *Escherichia coli* growth and (C) erythrocyte integrity in comparison to a negative control (20 mM MES pH 5.5). Statistical test: Kruskal-Wallis rank sum tests; n = 3 (bacterial growth, erythrocyte integrity), n = 6 (insect cell viability).

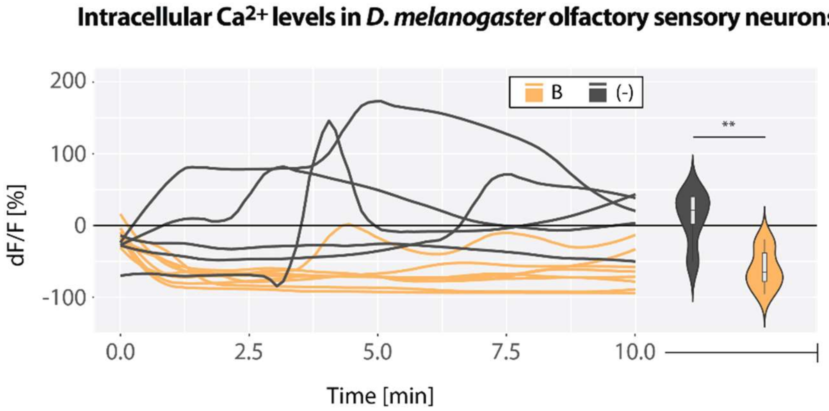


**Figure S3.** Calcium imaging of *Drosophila melanogaster* antennal lobes after treatment with fraction B. Changes of intracellular calcium concentration (represented by dF/F) in *Drosophila melanogaster* olfactory sensory neurons after treatment with fraction B. (-) = 20 mM MES + 1 M NaCl (pH 5.5). Violin plots represent the fluorescence intensity after 10 min. Statistical test: Kruskal-Wallis test (*$p \leq 0.05$; **$p \leq 0.01$; ***$p \leq 0.001$). Boxplots within the violin plots represent median (line), interquartile range (box) and data range (whiskers).

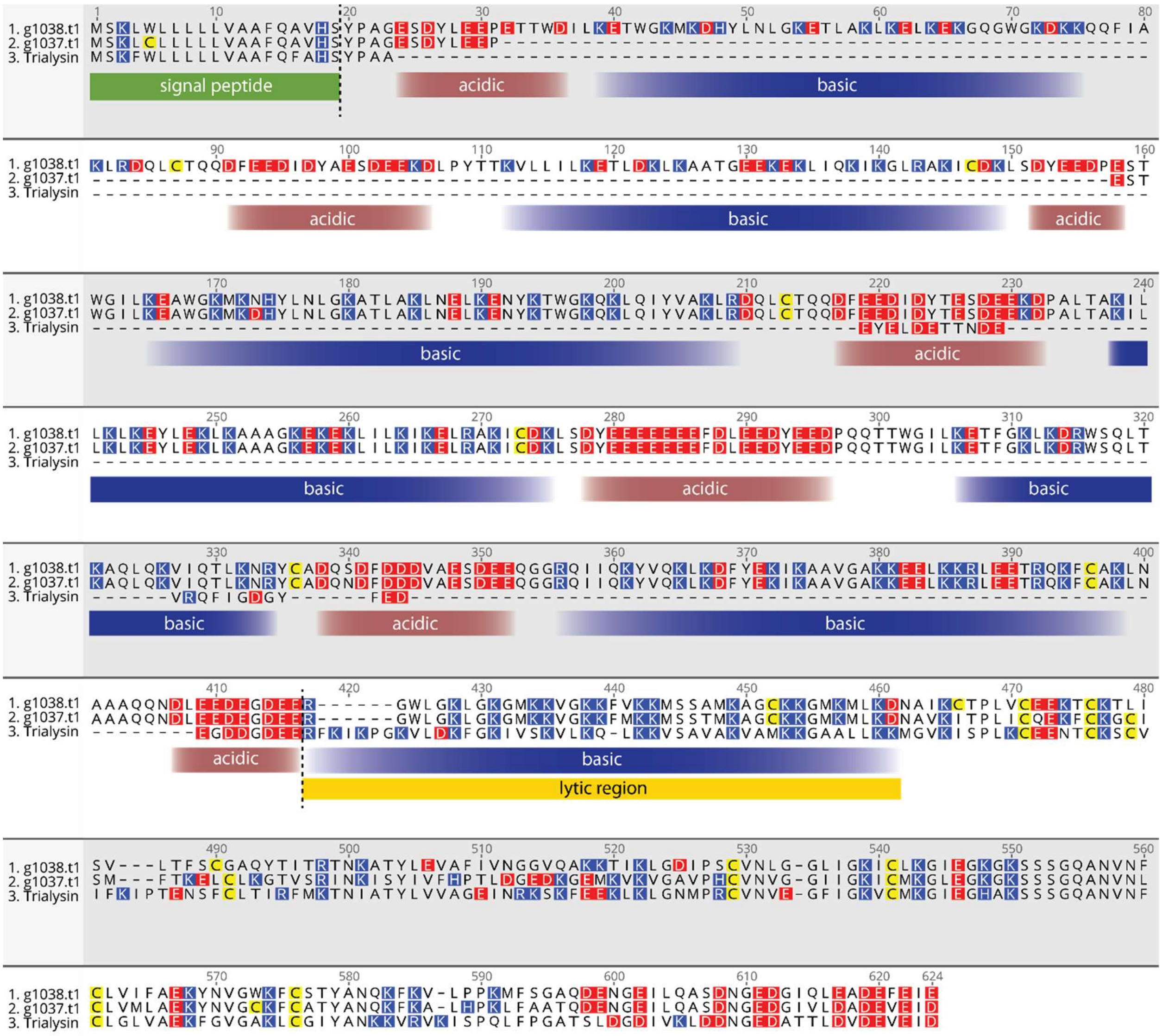


**Figure S4.** Alignment of *Psytalla horrida* redulysins g1038.t1, g1037.t1 and trialysin from *Triatoma infestans* (Genbank accession: AAL82381.1). Acidic and basic amino acids are highlighted in red and blue, respectively. Cysteine residues are highlighted in yellow. The signal peptide sequence is marked in green and the lytic region according to Amino et al. [11]in yellow. Dotted vertical lines represent the putative restriction sites after the signal peptide and pro-peptide.

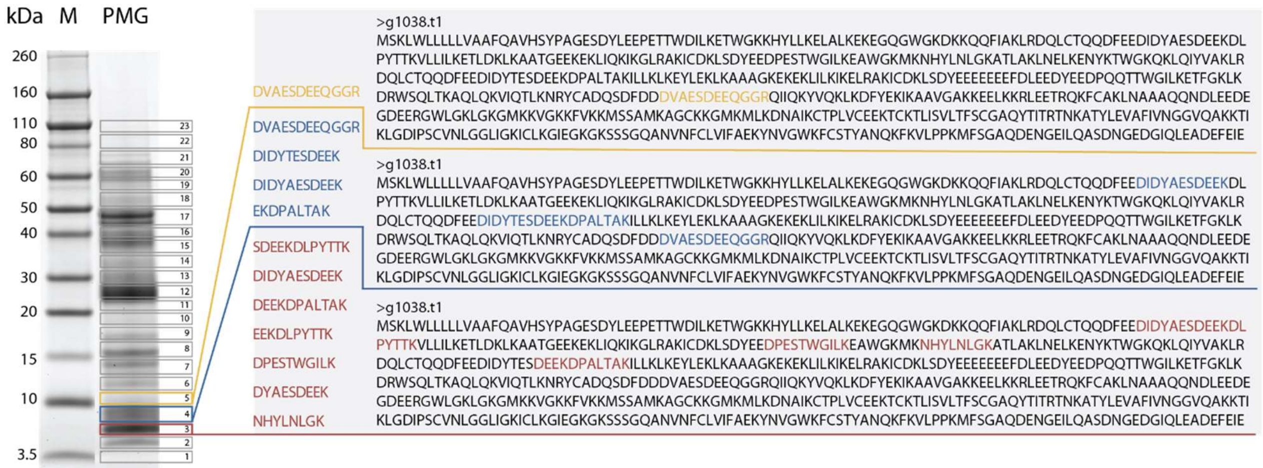


**Figure S5.** Redulysin g1038.t1 (contig Phor_Comb_C9529) identified in the LC-MS/MS analysis of *Psytalla horrida* PMG venom conducted in Fischer et al. [7]. The numbered boxes indicate the bands that were cut out for analysis. The colored boxes highlight the bands where redulysin g1038.t1 (Phor_Comb_C9529) was detected. The colored sequences represent

the peptides that were detected in the respective protein band and matched the amino acid sequence of g1038.t1. PMG = PMG extract; M = molecular weight marker. Details on methods of the transcriptomic and proteomic analysis are described in Fischer et al. [7].

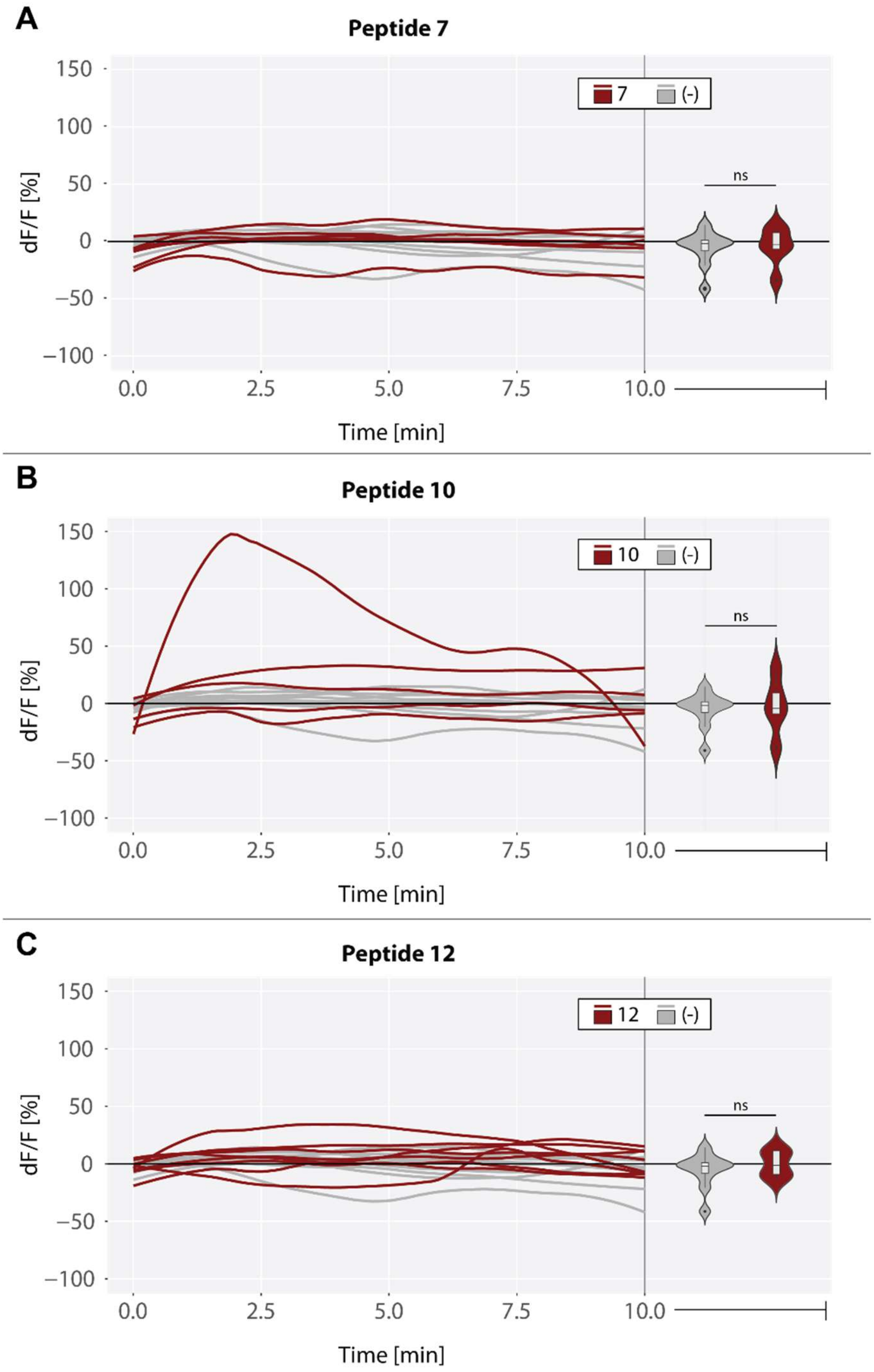


**Figure S6.** Calcium imaging of *Drosophila melanogaster* antennal lobes after treatment with 100 μM of selected redulysin peptides. Changes of intracellular calcium concentration (represented by dF/F) after treatment with peptide 7 (**A**), peptide 10 (**B**) or peptide 12 (**C**). (-) = 20 mM MES pH 5.5. Violin plots represent the fluorescence intensity after 10 min. Statistical test: Kruskal-Wallis test ($\alpha$ = 0.05). Boxplots within the violin plots represent median (line), interquartile range (box) and data range (whiskers).

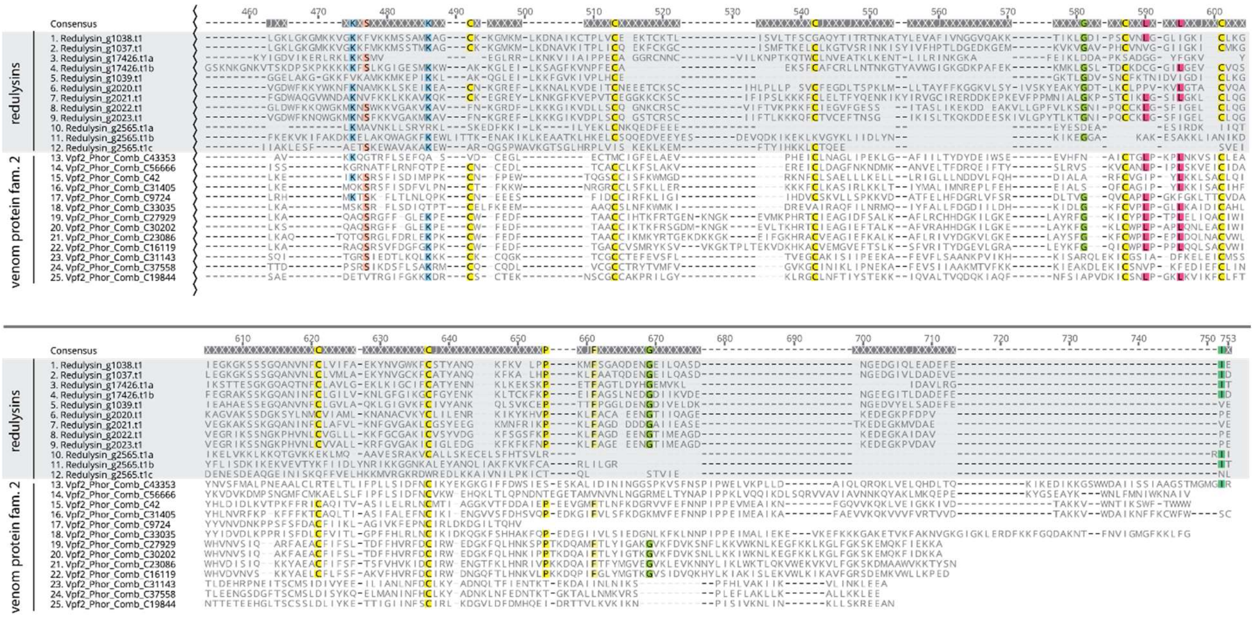

**Figure S7.** Alignment of the C-terminal region of the *Psytalla horrida* redulysins and venom family 2 proteins. Residues with > 50% agreement to the consensus are highlighted.


Supplementary references

1. Amino, R.; Martins, R.M.; Procopio, J.; Hirata, I.Y.; Juliano, M.A.; Schenkman, S. Trialysin, a novel pore-forming protein from saliva of hematophagous insects activated by limited proteolysis. *J. Biol. Chem.* **2002**, *277*, 6207–6213.
2. Fischer, M.L.; Wielsch, N.; Heckel, D.G.; Vilcinskas, A.; Vogel, H. Context-dependent venom deployment and protein composition in two assassin bugs. *Ecol. Evol.* **2020**, *10*, 9932–9947.
